# Systemic lupus erythematosus is associated with an increased risk of cervical artery dissection

**DOI:** 10.1038/s41598-025-85655-2

**Published:** 2025-01-07

**Authors:** Robert J. Trager, Benjamin P. Lynn, Anthony N. Baumann, Eric Chun-Pu Chu

**Affiliations:** 1 Connor Whole Health, University Hospitals Cleveland Medical Center, 11100 Euclid Ave, Cleveland, 44106 OH USA; 2 Department of Family Medicine and Community Health, Case Western Reserve University School of Medicine, Cleveland, OH USA; 3 Department of Biostatistics and Bioinformatics Clinical Research Training Program, Duke University School of Medicine, Durham, NC USA; 4 Los Gatos Sports Chiro, Los Gatos, CA USA; 5 College of Medicine, Northeast Ohio Medical University, Rootstown, OH USA; 6 Department of Rehabilitation Services, University Hospitals, Cleveland, OH USA; 7 New York Medical Group, EC Healthcare, Kowloon, Hong Kong

**Keywords:** Neurology, Rheumatology, Risk factors

## Abstract

Limited evidence suggests that autoimmune diseases are associated with an increased risk of cervical artery dissection (CeAD). We hypothesized individuals with systemic lupus erythematosus (SLE) would have an increased risk of CeAD following SLE diagnosis compared to matched non-lupus controls. We queried a de-identified United States electronic medical records network (TriNetX, Inc.) for individuals aged 10 and older from 2012 to 2020, for two cohorts: (1) SLE and (2) non-lupus controls, excluding those with prior CeAD. We used propensity matching to control for confounding variables and calculated the risk ratio (RR) for CeAD occurring over four years’ follow-up, secondarily exploring cumulative incidence. After matching, both cohorts contained 77,008 patients, who were mostly female (89%). The incidence and risk of CeAD was significantly greater among those with SLE compared to matched non-lupus controls [95% CI] (0.08% vs. 0.04%; RR = 2.33 [1.49;3.66]; *P* < 0.0001). These findings support the hypothesis that SLE is a risk factor for CeAD. Additional research is needed to identify the mechanisms that may underly the SLE-CeAD association and examine the potential association between other autoimmune diseases and CeAD.

**Supplementary Information:**

The online version contains supplementary material available at 10.1038/s41598-025-85655-2.

## Introduction

Systemic lupus erythematosus (SLE) is a chronic, multisystem autoimmune disorder involving the production of autoantibodies which lead to inflammation and tissue damage^1^. Limited research has suggested that autoimmune diseases, such as SLE, may be a risk factor for spontaneous cervical artery dissection (CeAD)^2–4^, a condition involving a tear and hematoma within the wall of the vertebral or carotid artery^5^. The incidence of SLE increases during adolescence, reaching up to 10 per 100,000 person-years in adults^6^. CeAD has an incidence of 8.9 per 100,000 person-years and accounts for 15–25% of strokes in adults under age 50^7^.

Specific etiologies of CeAD are recognized including trauma^8^ and iatrogenic causes such as spine surgery^9^. Several risk factors for CeAD have also been identified, albeit with varying degrees of certainty. Better-established risk factors include fibromuscular dysplasia and other genetic conditions affecting collagen^8,10^, hypertension^11,12^, migraine without aura^13^, pregnancy^14,15^, and respiratory infections^16–19^ (apart from coronavirus disease 2019^20^). Others potential risk factors like diabetes mellitus^11,21^, autoimmune thyroiditis^3,4^, hyperlipidemia^11,12^, fluoroquinolones^22^, oral contraceptives^16,23,24^, alcohol intake^11,25,26^, and tobacco use^12^ have conflicting or only preliminary evidence to date. Additionally, socioeconomic factors may influence whether patients undergo testing to identify CeAD^16,27^, highlighting the need for carefully designed studies to examine these risk factors.

Case-control studies have also revealed heightened inflammatory markers in individuals with CeAD compared to non-CeAD controls. Notably, elevations in C-reactive protein, erythrocyte sedimentation rate, and white blood cell counts have been observed^28–31^. While these findings suggest a potential underlying inflammatory mechanism for CeAD, the mediating factors remain unclear. Accordingly, some researchers have suggested that autoimmune disease may play a role, given these conditions can induce inflammation^2,4^. For example, one case-control study involving 215 CeAD cases found that 12.6% of individuals with CeAD had an autoimmune disease, compared to only 0.9% of age- and sex-matched population controls^4^. Therefore, while a direct link between specific autoimmune diseases and CeAD is yet to be confirmed, prior research indicates that further investigation into this relationship is warranted.

Individuals with SLE have an increased risk of several cardiovascular diseases, including myocardial infarction, stroke, atherosclerosis, and peripheral vascular disease^32^. However, little research has examined the potential association between SLE and arterial dissection of any type. One retrospective cohort study found that those with SLE had an increased risk of aortic dissection^33^, while a case-control study found a positive association between SLE and a potential precursor of aortic aneurysm^34^. In addition, several case reports have described spontaneous coronary artery dissection^35^ and CeAD occurring in those with SLE^36–41^, necessitating further research.

Several pathogenic mechanisms involved in SLE leading to vascular complications could also possibly increase the risk of CeAD. In general, those with SLE are more likely to have hypercoagulability, and occasionally develop vasculitis^42^. Additionally, chronic inflammation and cross-reactivity of antibodies and complement proteins with arterial walls may lead to arterial wall dysfunction, stiffening, and/or degradation^2,34,43^. Illustratively, individuals with SLE have greater common carotid artery intima-media thickness compared to matched controls^44^. SLE is also associated with genetic variation in collagen^45^, and generalized joint hypermobility^46^, both factors which have been associated with CeAD^47^.

Considering the precursory evidence suggesting SLE could increase the risk of CeAD, we further examined this possible association via a retrospective cohort design to better understand patient risk profiles. We hypothesized that individuals first diagnosed with SLE would have an increased risk of CeAD compared to propensity-matched non-lupus controls, assessed over a four-year follow-up window.

## Materials and methods

### Study design

We use a retrospective cohort design to enable us to calculate the incidence of CeAD after SLE diagnosis. Patients were included from 10 to 4 years prior to the query date (November 4, 2024), to maximize sample size. We obtained data from the US TriNetX collaborative research network^48^, which represents 96 healthcare organizations and more than 134 million de-identified patients. This network includes academic and other large medical centers, community hospitals, and their associated ambulatory clinics, which contribute longitudinal, routinely collected electronic health record data. The TriNetX platform standardizes data using common terminologies such as the International Classification of Disease, 10th Revision (ICD-10) diagnosis codes. TriNetX employs data quality checks, including assessment of conformance to standard terminologies, consistency across data domains, and completeness^49^. TriNetX maintains patient privacy through several means including de-identification, privacy-preserving record linkage, and contractual protections. We used the TriNetX platform online query builder to define study cohorts^48,50^. This tool retrieved and counted patients who met the eligibility outlined in the Methods section and Supplemental File. Diagnoses such as SLE and CeAD included in the analysis represent those appended by clinicians within the participating healthcare organizations as part of real-world clinical practice.

This study was determined ‘Not Human Subjects Research’ by the University Hospitals Institutional Review Board (Cleveland, Ohio, US, STUDY20230507). Due to the retrospective nature of the study, the University Hospitals Institutional Review Board waived the need of obtaining informed consent. All methods were performed in accordance with the relevant guidelines and regulations. We present our findings in accordance with the Strengthening the Reporting of Observational Studies in Epidemiology guideline^51^. A graphical depiction of the study design is available (Supplemental File Figure S1).

### Eligibility criteria

We included patients age 10 years and older and divided them into two cohorts: (1) SLE; patients included at the first instance of SLE diagnosis (ICD-10: M32) and (2) non-lupus; patients having a pediatric or adult medical examination (ICD-10: Z00.1 and Z00.0, respectively) and without a diagnosis of lupus, SLE, lupus anticoagulant syndrome, systemic connective tissue disorder, inflammatory polyarthropathy, antiphospholipid syndrome, or presence of antinuclear, anti-smith, or anti-double-stranded DNA antibodies. To maximize data completeness, we required a healthcare visit between one day and three years prior to the index date of inclusion. To minimize loss to follow-up, we required either a healthcare visit or recorded status of ‘deceased’ between one month and four years’ follow-up. We avoided including those with only cutaneous lupus in our SLE cohort, as this condition may have a different cardiovascular risk profile with respect to CeAD. However, in the current design, patients with SLE were eligible to also have cutaneous lupus, accounting for the common overlap between these conditions^52^. We excluded patients with previous dissection, aneurysm, surgery, or trauma of the cervical arteries as well as those with external causes of morbidity (e.g., motor vehicle collisions), spine surgery, or intubation within the month preceding and including the index date. We used a 30-day washout for external causes of morbidity considering the mean time to diagnosis of CeAD is approximately 9 days (standard deviation [SD] = 12 days)^53^. Therefore, a 30-day period would effectively exclude most patients with trauma-related CeAD from entering the study at baseline.

To minimize misclassification related to suspected diagnoses of SLE, we required patients in the SLE cohort to have at least two additional instances of the ICD-10 diagnosis (M32) following the index date^54^. Additionally, we excluded patients in the non-SLE who were appended an SLE diagnosis at any point during follow-up. Selection criteria are detailed in the Supplemental File Table S1.

### Variables

We used propensity score matching to reduce bias, matching variables present within one year preceding and including the index date of inclusion which have an association with CeAD (Supplemental File Table S2). To address potential temporal bias related to between-cohort differences in the years of cohort entry, we matched patients by age at the index date and current age (at the time of our query). This approach aimed to minimize confounding related to the increasing incidence of CeAD over time, possibly related to improved detection^7^.

### Outcomes

Our primary outcome included a composite of CeAD types including carotid artery dissection (ICD-10: I77.71) and vertebral artery dissection (ICD-10: I77.74)^18,55,56^. Considering these conditions are rare, pooling them increased our ability to identify differences between cohorts. We ascertained CeAD occurrences over a four-year follow-up to maximize our ability to identify sufficient events, commencing the day after the index date. Considering SLE is a slow progressive disease^57^, we did not expect an immediate impact on CeAD.

As a secondary analysis, we examined the cumulative incidence of CeAD. If there were more than 10 cases of vertebral artery dissection and carotid artery dissection in each cohort, we calculated the RR for these outcomes separately. To better characterize treatments administered in our SLE cohort and provide markers of generalizability, we will examine the proportion of patients prescribed pharmacological treatments commonly indicated for SLE. This will include corticosteroids for systemic use (Anatomical Therapeutic Chemical [ATC]: H02), hydroxychloroquine (RxNorm: 5521), immunosuppressants (ATC: L04), and/or antineoplastic agents (ATC: L01).

### Statistical methods

We used built-in functions of the TriNetX statistical platform to compare baseline characteristics and perform propensity score matching. We compared baseline variables using standardized mean difference (SMD) values, with a threshold of less than 0.2 indicating successful matching^58,59^. TriNetX uses Python (scikit-learn version 1.3, Python Software Foundation, Delaware, US) to apply logistic regression to pooled covariate matrices when calculating propensity scores. Propensity scores of 1 indicate the greatest likelihood of being in the non-lupus cohort. Greedy nearest-neighbor matching is performed using with a 1:1 ratio and caliper of 0.1 pooled SD. To calculate risk ratios (RRs), we divided the incidence of CeAD in the SLE cohort by the incidence in the non-lupus cohort, with statistical significance evaluated at *P* < 0.05. We used R and R studio (version 4.2.2, Vienna, AT^60^) and the ggplot2 package^61^ to graph covariate balance, follow-up metrics, total and cumulative incidence, and propensity score density. To further assess data quality and markers of matching adequacy, we explored follow-up metrics and negative control outcomes, interpreting SMD (< 0.2^58,59^) and RR (0.8 ≥ RR ≤ 1.2^62^) values as balanced between cohorts. Total person-years of follow-up for each cohort were calculated by summing the follow-up times, in years, for all patients within each cohort. Patients were censored the day after the last clinical fact appearing in their record yet were not censored for mortality. Unless otherwise specified above, categorical outcomes were compared using Pearson’s chi-squared test while continuous variable outcomes were compared using an independent samples t-test.

### Required study size

We initially estimated a total required sample size of 202,064 for a follow-up duration of two years, using GPower (Version 3.1.9.6, Kiel University, DE) via a z-test to discern a difference in incidence proportion between cohorts (0.0002 vs. 0.0005^7,33^) with an α-error of 0.05, allocation ratio of one, two tails, and power of 0.95. Upon peer reviewer feedback, we lengthened the follow-up window to four years. Upon revisiting our initial calculations, we found that doubling the expected total incidences approximately halved the required total sample size to 100,994 patients.

## Results

### Patients

Before propensity matching there were 80,331 patients in the SLE cohort and 1,321,616 non-lupus controls. After matching, each cohort had 77,008 patients, with a female predominance (89%). Before matching, patients in the SLE cohort had an older mean age at the index date and query date, were more often female, and had a greater proportion of various comorbidities and medications (SMD > 0.2, Table 1). After matching, all covariates were adequately balanced (SMD < 0.2). Funnel diagrams illustrating the impact of selection criteria on the cohort sizes are available (Supplemental File Figure S2 and Figure S3).

**Table 1:** Baseline patient characteristics before and after matching.

Variable	Before matching			After matching		
Variable (n (%) or mean (SD))	SLE	Non-lupus controls	SMD	SLE	Non-lupus controls	SMD
N	80,331	1,321,616	NA	77,008	77,008	NA
Age at Index	47.2 (16.0)	41.1 (20.7)	0.331	47.0 (16.0)	48.9 (16.8)	0.114
Age at Query (Current Age)	55.1 (16.0)	48.1 (20.5)	0.381	54.9 (16.0)	56.6 (16.8)	0.105
Female	69,645 (87%)	769,636 (58%)	0.672	68,696 (89%)	68,316 (89%)	0.016
Male	7924 (10%)	551,511 (42%)	0.782	7922 (10%)	8223 (11%)	0.013
Acute Upper Respiratory Infections	6546 (8%)	150,557 (11%)	0.109	6336 (8%)	5765 (7%)	0.028
Adverse Socioeconomic & Psychosocial Factors	693 (1%)	17,186 (1%)	0.042	674 (1%)	611 (1%)	0.009
Alpha-1-Antitrypsin Deficiency	22 (0%)	188 (0%)	0.009	20 (0%)	20 (0%)	0.000
Antihypertensives	4692 (6%)	43,190 (3%)	0.124	4289 (6%)	4301 (6%)	0.001
Antimigraine Agents	2469 (3%)	21,353 (2%)	0.096	2372 (3%)	2354 (3%)	0.001
Aortic Aneurysm and Dissection	389 (0%)	4455 (0%)	0.023	365 (0%)	411 (1%)	0.008
Arterial Fibromuscular Dysplasia	116 (0%)	80 (0%)	0.050	63 (0%)	57 (0%)	0.003
Autoimmune Thyroiditis	1336 (2%)	5071 (0%)	0.127	1195 (2%)	1252 (2%)	0.006
Beta Blockers/Related	12,455 (16%)	98,173 (7%)	0.256	11,789 (15%)	12,701 (16%)	0.032
Contraceptives, Systemic	2385 (3%)	64,231 (5%)	0.098	2345 (3%)	1973 (3%)	0.029
Diabetes Mellitus	8279 (10%)	97,746 (7%)	0.103	7852 (10%)	8720 (11%)	0.036
Diseases of Arteries, Arterioles and Capillaries	8469 (11%)	25,768 (2%)	0.361	7248 (9%)	7633 (10%)	0.017
Ehlers-Danlos Syndromes	165 (0%)	501 (0%)	0.048	130 (0%)	216 (0%)	0.024
Family History of Ischemic Heart Disease	1695 (2%)	26,559 (2%)	0.007	1618 (2%)	1699 (2%)	0.007
Homocystinuria	162 (0%)	388 (0%)	0.051	134 (0%)	213 (0%)	0.022
Hyperlipidemia, Unspecified	10,332 (13%)	167,713 (13%)	0.005	9873 (13%)	10,523 (14%)	0.025
Hypertensive Diseases	27,268 (34%)	278,855 (21%)	0.291	25,831 (34%)	26,885 (35%)	0.029
Migraine	5520 (7%)	39,775 (3%)	0.179	5116 (7%)	5168 (7%)	0.003
Osteogenesis Imperfecta	≤ 10 (0%)	112 (0%)	0.004	≤ 10 (0%)	≤ 10 (0%)	0.000
Other Specified Congenital Malformation Syndromes	97 (0%)	840 (0%)	0.019	87 (0%)	100 (0%)	0.005
Overweight And Obesity	8468 (11%)	138,201 (10%)	0.003	8157 (11%)	7994 (10%)	0.007
Pregnancy, Childbirth and The Puerperium	2981 (4%)	19,216 (1%)	0.143	2828 (4%)	3033 (4%)	0.014
Quinolones	7288 (9%)	58,526 (4%)	0.186	6866 (9%)	6973 (9%)	0.005
Substance Use Disorder	7522 (9%)	89,684 (7%)	0.095	7070 (9%)	7937 (10%)	0.038
Tobacco Use	1049 (1%)	30,268 (2%)	0.074	992 (1%)	1197 (2%)	0.022

Abbreviations: standard deviation (SD), standardized mean difference (SMD), systemic lupus erythematosus (SLE). Cell counts fewer than 10 are rounded up to 10 for de-identification purposes.

### Data quality and propensity score diagnostics

The mean number of data points (i.e., facts; diagnoses, test results) per patient per cohort was sufficient (SLE: 5,215; non-lupus: 1,644). Following matching, propensity score densities closely overlapped (Supplemental File Figure S4), SMD values were adequate (Supplemental Figure S5), and the proportion of those with an unknown sex was 1% in both cohorts (SMD = 0.014). Comparing the SLE to non-lupus control cohort over the four-year follow-up, the likelihoods of negative control outcomes were balanced, including having an encounter for delivery (i.e., birth) (1.3% vs. 1.5%; RR = 0.84) and colonoscopy (9.1% vs. 10.9%; RR = 0.83). After matching, the mean follow-up duration in days was not meaningfully different between cohorts [SD] (SLE: 1,317 [335]; non-lupus controls: 1,341 [319]; SMD = 0.073) while the median follow-up was identical [interquartile range] (both cohorts: 1,460 [0]). The minimum and maximum follow-up was 30 and 1460 days, respectively, for both cohorts. The total person-years of follow-up observation were similar (SLE: 277,644; non-lupus controls: 282,671), differing by 1.8%. Additional details regarding follow-up are shown in Supplemental File Figure S6. The SLE cohort had a greater incidence and risk of a ‘deceased’ status (4.8% vs. 2.6%, RR = 1.82 [1.73,1.92]; *P* < 0.0001) and mean number of follow-up visits [SD] (71.5 [80.9] vs. 54.1 [60.3]; *P* < 0.0001) compared to non-lupus controls. Despite the greater mean number of follow-up visits in the SLE cohort, the similar follow-up duration, adequate markers of data density and completeness, balanced negative control outcomes, and balanced demographics and unknown variables suggest there were no meaningful between-cohort differences in covariate balance, data density, completeness, or attrition.

### Primary outcome

**Fig. 1:**
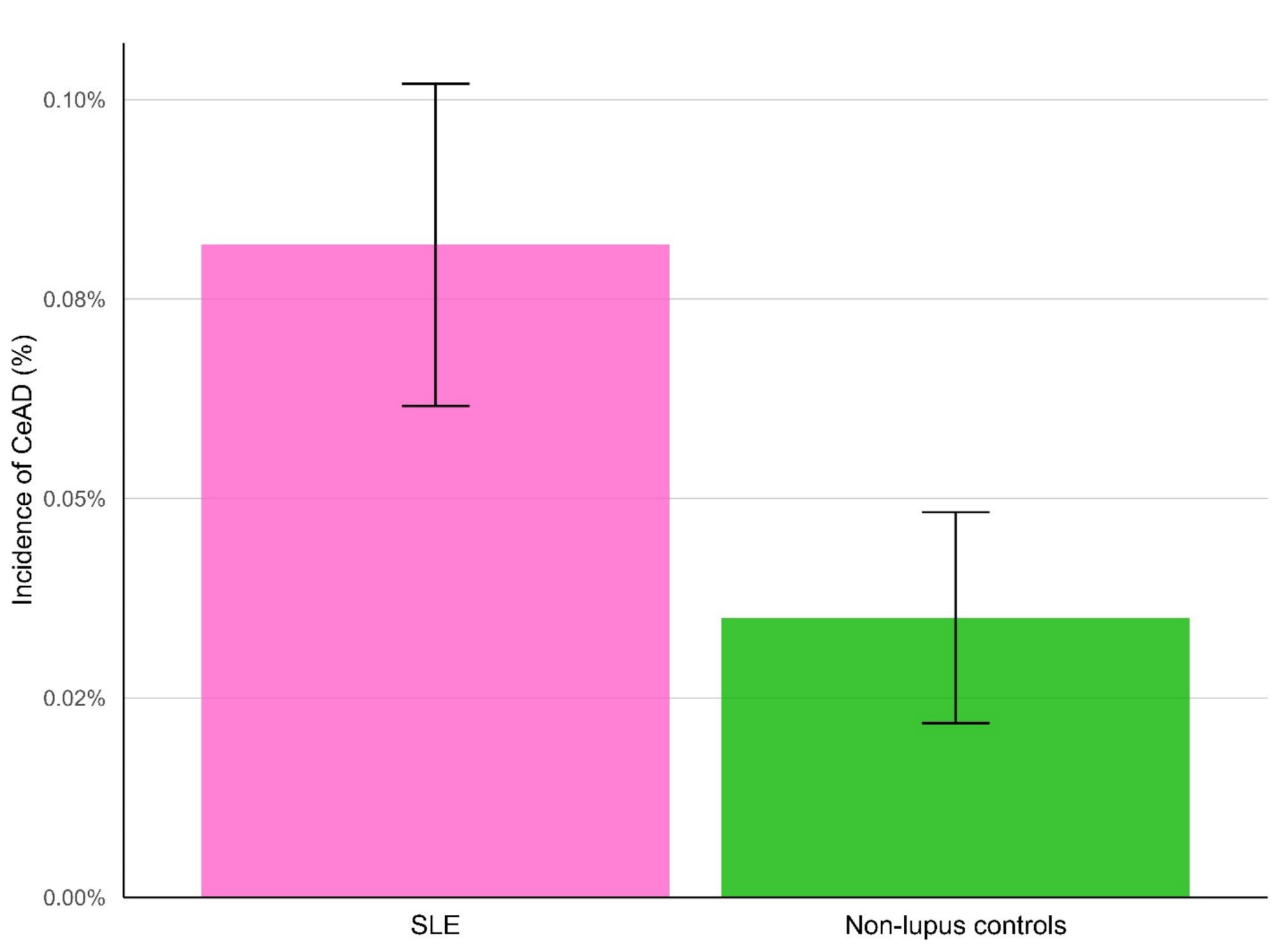
Total incidence of cervical artery dissection (CeAD) over the four-year follow-up window. Incidences of CeAD are shown for the systemic lupus erythematosus cohort (SLE; pink) and non-lupus controls (green). Brackets indicate 95% confidence intervals.

After matching, the incidence and risk of CeAD was significantly greater among those with SLE compared to matched non-lupus controls [95% CI] (0.08% vs. 0.04%; RR = 2.33 [1.49;3.66]; *P* < 0.0001), as presented in Table 2; Fig. 1.

**Table 2:** Incidence and risk ratio of cervical artery dissection.

	Before matching		After matching*	
Measure	SLE	Non-lupus controls	SLE	Non-lupus controls
Total patients	80,331	1,333,666	77,008	77,008
Total person-years of observation	289,742	4,739,595	277,644	282,671
CeAD n (%)	66 (0.08%)	361 (0.03%)	63 (0.08%)	27 (0.04%)
CeAD n per 100,000 person-years	20.6	6.8	20.5	8.8
CeAD risk ratio (95% CI; *P*)	3.04 (2.33, 3.95; *P* < 0.001)	Reference	2.33 (1.49, 3.66; *P* < 0.001)	Reference

Abbreviations: Cervical artery dissection (CeAD)

*Data used for primary outcome

### Secondary outcomes

**Fig. 2:**
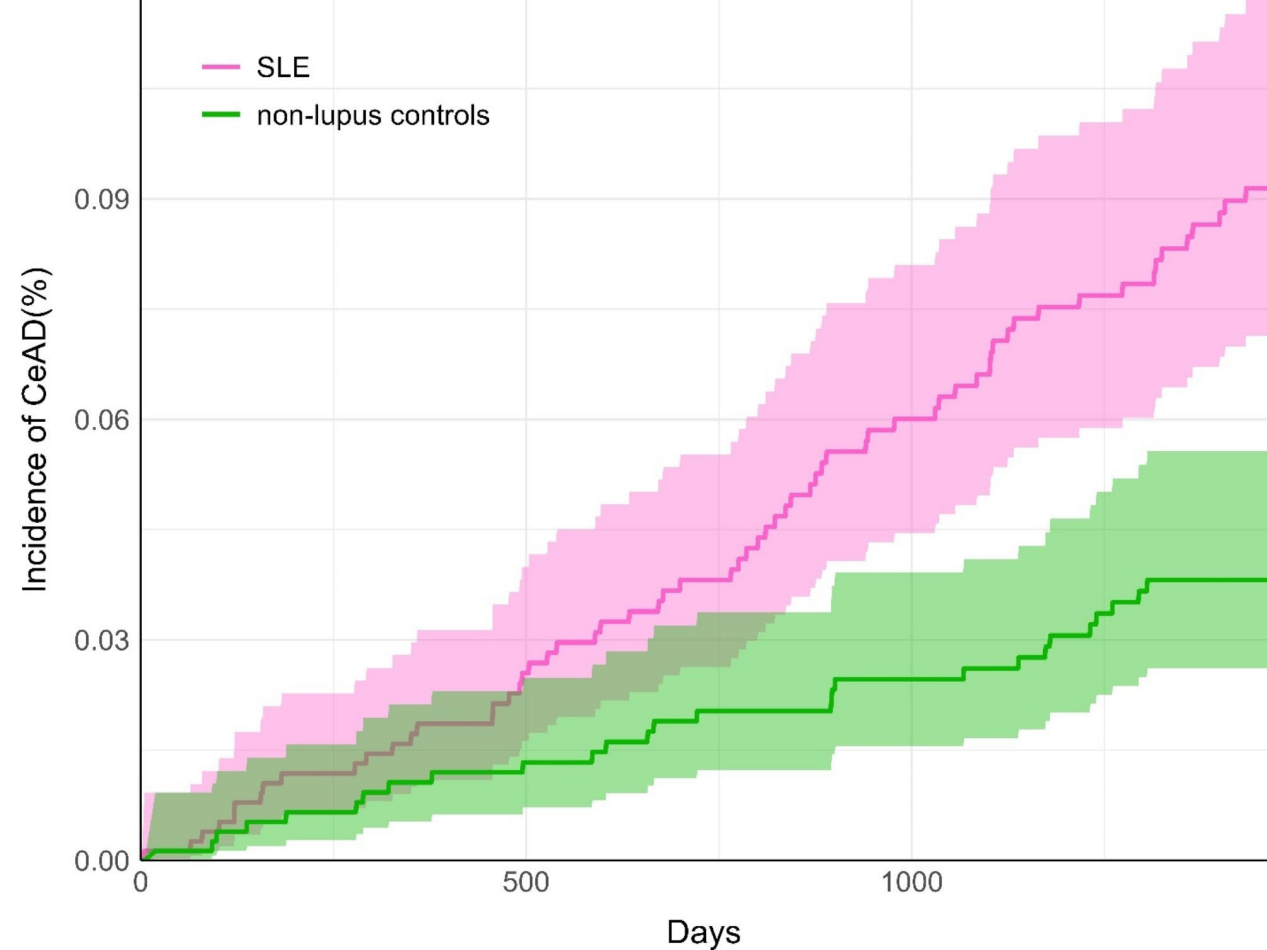
Cumulative incidence of cervical artery dissection (CeAD). Incidences of CeAD in the systemic lupus erythematosus cohort (SLE; pink) and non-lupus controls (green) are illustrated over the four-year follow-up period (1460 days). Shaded regions indicate 95% confidence intervals.

After matching, evaluation of cumulative incidence highlighted a gradual relative increase in CeAD incidence among those with SLE compared to non-lupus controls (Fig. 2). By the end of the four-year follow-up window, there was a clear separation between the cohorts’ incidences and 95% confidence intervals.

After matching, the incidence and risk of vertebral artery dissection was greater among those with SLE compared to matched non-lupus controls, although this finding was not statistically significant [95% CI] (23 vs. 16 patients; 0.030% vs. 0.021%; RR = 1.44 [0.76,2.72]; *P* = 0.2623). The incidence and risk of carotid artery dissection was significantly greater among those with SLE compared to matched non-lupus controls [95% CI] (43 vs. 11 patients; 0.056% vs. 0.014%; RR = 3.91 [2.02,7.58]; *P* < 0.0001). Total and cumulative incidence plots for vertebral and carotid artery dissection are also available (Supplemental File Figure S7, Figure S8, Figure S9, and Figure S10).

After matching, the proportion and mean prescription count for medications commonly used to treat SLE was substantial over the four-year follow-up in the SLE cohort. This included corticosteroids [SD] (74%; mean = 13.4 [26.0]), hydroxychloroquine (55%; mean = 12.7 [24.8]), immunosuppressants (34%; mean = 14.2 [27.3]), antineoplastic agents (25%; mean = 8.7 [17.8]).

## Discussion

The present findings support the hypothesis of a statistically significant increase in risk of CeAD among individuals with SLE compared to matched non-lupus controls, contributing to our unknowledge of risk factors for CeAD and the risk profile of SLE. CeAD has potentially meaningful and chronic sequelae, and in general, 29–65% of patients with CeAD develop a stroke^63,64^. Although we observed a significant SLE-CeAD association; secondary outcomes were mixed, with vertebral artery dissection showing a non-significant association and carotid artery dissection showing a significant, positive association. Considering these subsets of CeAD are rarer than CeAD itself and study was not powered to examine these pathologies individually, these estimates should be interpreted cautiously and warrant further corroboration in future matched studies.

Direct comparisons with external data should also be made with caution due to potential differences in study populations (e.g., demographics, selection criteria). The mean age of diagnosis of SLE has been reported in the US as variably 42 years (SD = 18)^65^ to 46 years (SD = 17)^66^. Comparatively, our SLE cohort mean age was slightly greater (i.e., 47 years), yet still within the expected variance. This slight increase in age could be explained by our selection criteria, which required patients to have a preceding healthcare visit, and excluded individuals under age 10. Similarities between our results and those of previous studies help validate and contextualize our findings. In the present study, the CeAD incidences in the pre- and post-matching SLE cohorts (approximately 21 per 100,000 person-years) were greater than a previous estimate from the US general adult population of 8.9 per 100,000 person-years using data from 2017 to 2020^7^. Conversely, the incidences of CeAD in our non-lupus control cohort of 6.8 to 8.8 per 100,000 person-year are comparable to the previous value of 8.9 per 100,000^7^, therefore serving as a marker of validity of our study. The female predominance of our SLE cohort is expected for this condition^6^. Finally, the two most common medications used in those with SLE are hydroxychloroquine and corticosteroids^67^, which matches with our findings.

Our study highlights a significant association between SLE and CeAD, supporting previous research indicating that autoimmune diseases can elevate CeAD risk. For example, one case-control study found that patients with at least one autoimmune disease had 2.9 times greater odds of having a spontaneous cerebral or cervical arterial dissection^4^, while another found that CeAD patients had a significantly greater incidence of thyroid autoimmunity than non-CeAD controls^3^. Given that CeAD is often considered spontaneous^2^, our study underscores the need for further research to clarify the relationship between autoimmune conditions and CeAD,

The mechanisms underlying the observed association between SLE and CeAD remain unclear. First, our propensity matching strategy controlled for common comorbidities like diabetes and hypertension, making these factors unlikely to account for the observed positive SLE-CeAD association. However, several other factors related to SLE may account for the increased CeAD risk. Chronic inflammation and antibody cross-reactivity may contribute to endothelial dysfunction and cervical arterial wall thickening^2,34,43,44^. Our observed gradual time-dependent increase in CeAD incidence in SLE patients suggests a delayed risk, possibly linked to cumulative pathophysiological factors in SLE. Genetic factors, SLE-related hypercoagulability, and vasculitis may also play a role^2,42,68^.

Future research is needed to clarify the SLE-CeAD association. It is necessary to investigate whether the association is explained by elevated antibody titers, inflammatory markers, or other changes associated with SLE, including cytokines such as B-cell activating factor, which may be influenced by lifestyle factors^69^. Additionally, it remains unclear whether treatments for SLE mediate the positive association with CeAD. This information may help identify underlying mechanisms and inform preventive strategies for those at higher risk of CeAD. Finally, research should explore whether other autoimmune diseases, such as inflammatory bowel disease or psoriasis, also increase the risk of CeAD to determine if the observed association is specific to certain autoimmune disorders or represents a broad increase in risk.

Our findings may help identify patients at risk for CeAD. Currently, CeAD diagnosis presents a challenge due to its benign-appearing early symptoms, reflected by a mean time-to-diagnosis of 9 days (SD = 12)^53^. Considering that the risk of CeAD may be compounded when multiple signs, symptoms, or risk factors are present, such as headache, hypertension, and pregnancy^8,14^, the presence of SLE may further increase pre-test probability. This information may be most relevant to clinicians frequently encountering SLE patients or those at risk of CeAD, including primary care physicians, rheumatologists, chiropractors, and physical therapists^70^. Accordingly, an increased index of suspicion for CeAD would raise the necessity to refer for emergency care and/or consider vascular imaging to identify CeAD.

Strengths and limitations.

Strengths of this study include a large sample size, robust matching strategy, registered protocol, and multidisciplinary investigator team. Limitations should also be noted. As an observational study, unmeasured confounding may be present. It was not possible to adjust for baseline body mass index, which was inconsistently available among the study population during the covariate assessment window. There may be additional unmeasured genetic or socioeconomic variables that could affect CeAD likelihood. For the SLE cohort, we rely on a clinical diagnosis of SLE and were unable to corroborate this diagnosis via manual chart review, which increases the potential for misclassification bias. The positive predictive value (PPV) of the ICD-10 diagnosis of SLE is limited, and in one study in Estonia was estimated to be 59.7% (95%CI: 55.8, 63.4)^54^. The authors noted that the PPV is relatively lower with fewer instances of the diagnosis, and higher when there are more instances, specifically when a rheumatologist appends the diagnosis^54^. Given our inability to determine if patients had visited a rheumatologist, the PPV of our strategy remains unclear. The positive predictive value for ICD coding for CeAD has been reported to be 90%^19^, yet the overall accuracy of our outcome ascertainment is unclear. We were unable to thoroughly describe disease markers in the SLE cohort, such as C-reactive protein, erythrocyte sedimentation rate, or antibody titers, due to variability in testing timing and indications prior to the index date, poor representation of these tests, and a lack of data granularity that hindered interpretation. However, given the existing literature suggesting a potential inflammatory milieu among those with CeAD^28–31^, our findings indicate that these markers warrant further investigation to determine their potential role in mediating the SLE-CeAD association. Given the expected limitations in sample size, there may be heterogeneity within the SLE cohort in terms of disease severity or presentation. It is also possible that patients had a long lag time until SLE diagnosis, which could artificially decrease the SLE-CeAD association as undiagnosed SLE patients having CeAD would be excluded in the present methods. The present findings may only apply to those with SLE rather than cutaneous lupus. Due to the limitations in data granularity, we were unable to determine the exact proportion of patients under age 18. We observed a higher mortality in the SLE cohort, yet this is unlikely to explain our findings. Any potential bias from mortality would likely decrease the magnitude of the observed SLE-CeAD association rather than increase it. Finally, our observations derive from the United States and may not apply to other countries in which the incidence of SLE and/or CeAD may differ.

## Conclusion

Our study supports a statistically significant increase in the risk of CeAD among individuals with SLE compared to matched non-lupus controls. This improved understanding of SLE as a risk factor for CeAD may aid in the identification of CeAD in clinical settings. However, it is less clear on how this risk factor translates to either vertebral or carotid artery dissection individually. Further research is needed to clarify the mechanisms underlying the SLE-CeAD association and explore the potential mediating effects of inflammatory and autoimmune biomarkers and treatments. Additionally, future studies should examine whether other autoimmune diseases similarly increase CeAD risk.

## Electronic supplementary material

Below is the link to the electronic supplementary material.

Supplementary Material 1

## Data Availability

Minimal, aggregate, de-identified datasets used for our primary outcome, cumulative incidence, and propensity score density plots are available in a Figshare repository (10.6084/m9.figshare.26496586^71^).
