# Supplementary material for: Systemic lupus erythematosus is associated with an increased risk of cervical artery dissection
Source: Sci Rep. 2025 Jan 7;15:1194. doi: 10.1038/s41598-025-85655-2 (PMC11707269; doi:10.1038/s41598-025-85655-2)
Supplement: Supplementary file 1 — Supplementary Material 1 [file 41598_2025_85655_MOESM1_ESM.pdf]

# Supplemental File

## For: Systemic lupus erythematosus is associated with an increased risk of cervical artery dissection

*Robert J. Trager, Benjamin P. Lynn, Anthony N. Baumann, Eric Chun-Pu Chu*

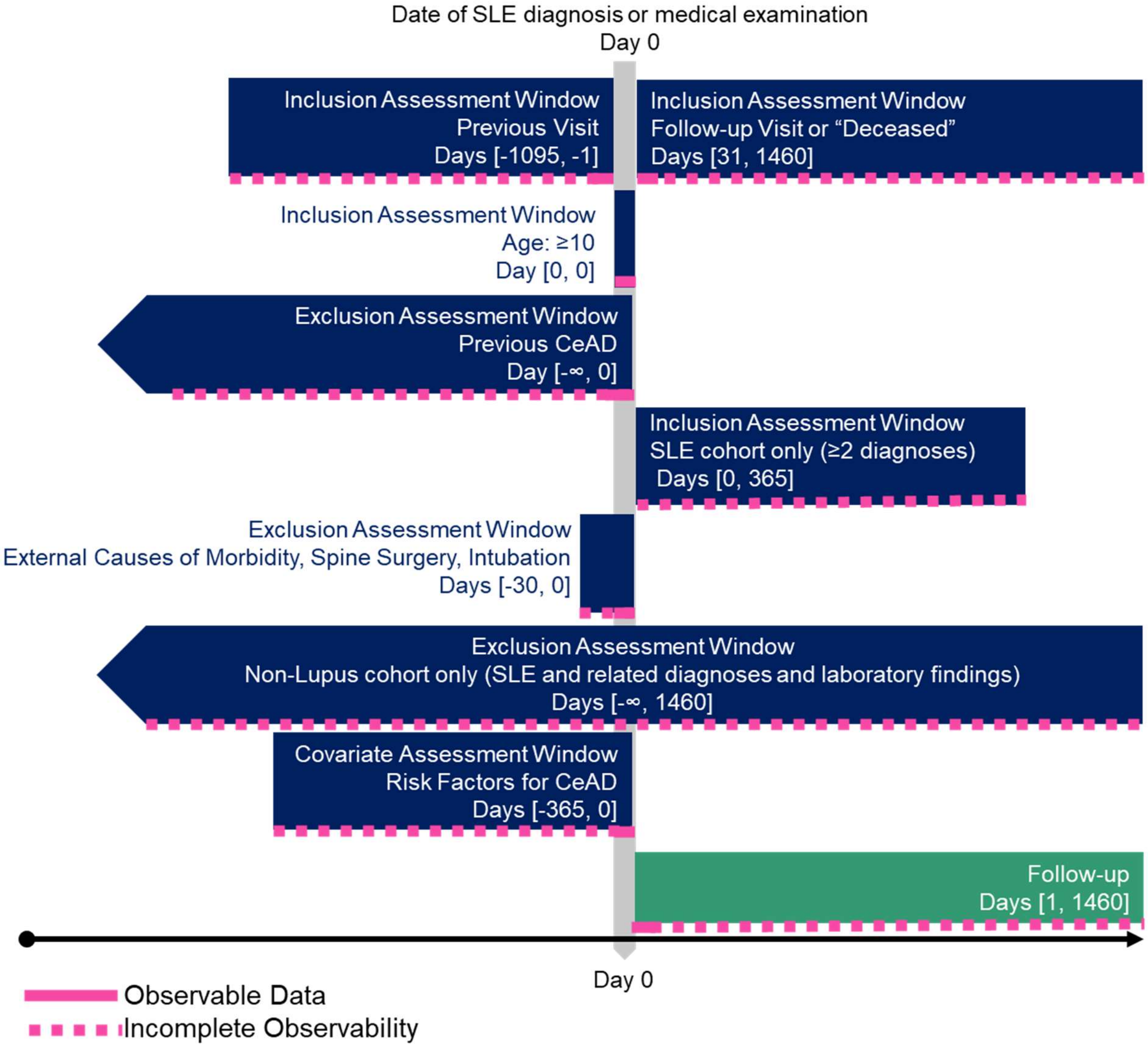


*Figure S1: Graphical depiction of study design. The vertical gray arrow indicates the index date (cohort entry date, day 0), which is the first diagnosis of systemic lupus erythematosus (SLE) or index medical examination. Text and boxes describe study selection criteria which were assessed during time windows ([#, #]) in the specified number of days in relation to the index date. Certain criteria spanned as long as preceding data were available per patient (-∞). Figure created by Robert J. Trager using a Creative Commons template from Wang et al [1].*

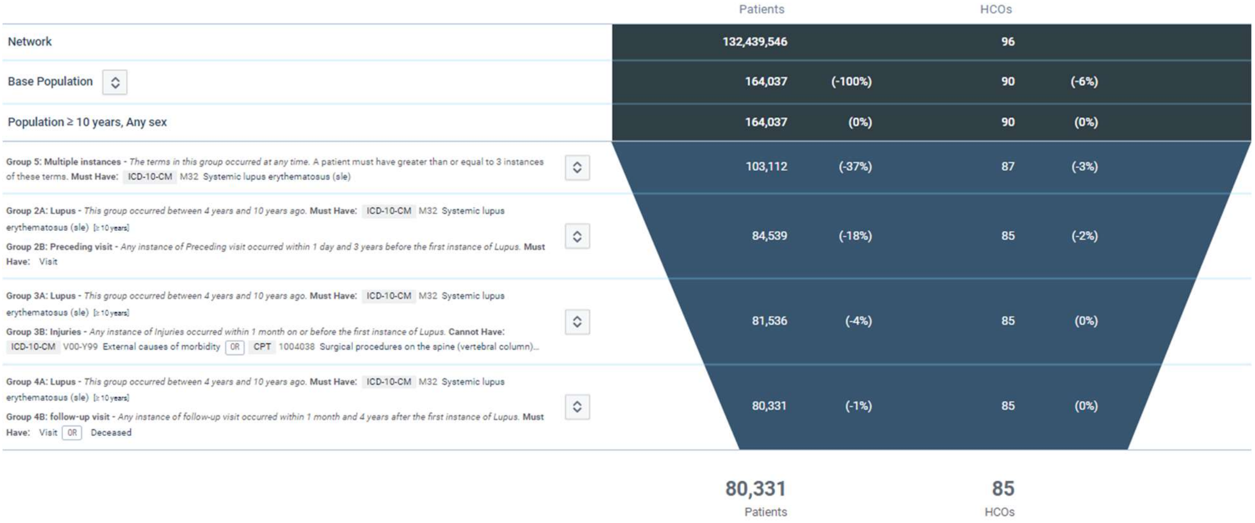


*Figure S2: Funnel diagram for the systemic lupus erythematosus cohort. The diagram illustrates the query groups described in the Supplemental File which are grouped according to similar time windows of assessment, indicating key International Classification of Diseases (ICD-10) codes, and ranked in terms of most to least impact on the sample size. The diagram illustrates the cohort's makeup prior to propensity matching and does not reflect the additional trimming that occurs when patients that do not match are discarded from the cohort. The number of healthcare organizations (HCOs) is also illustrated.*

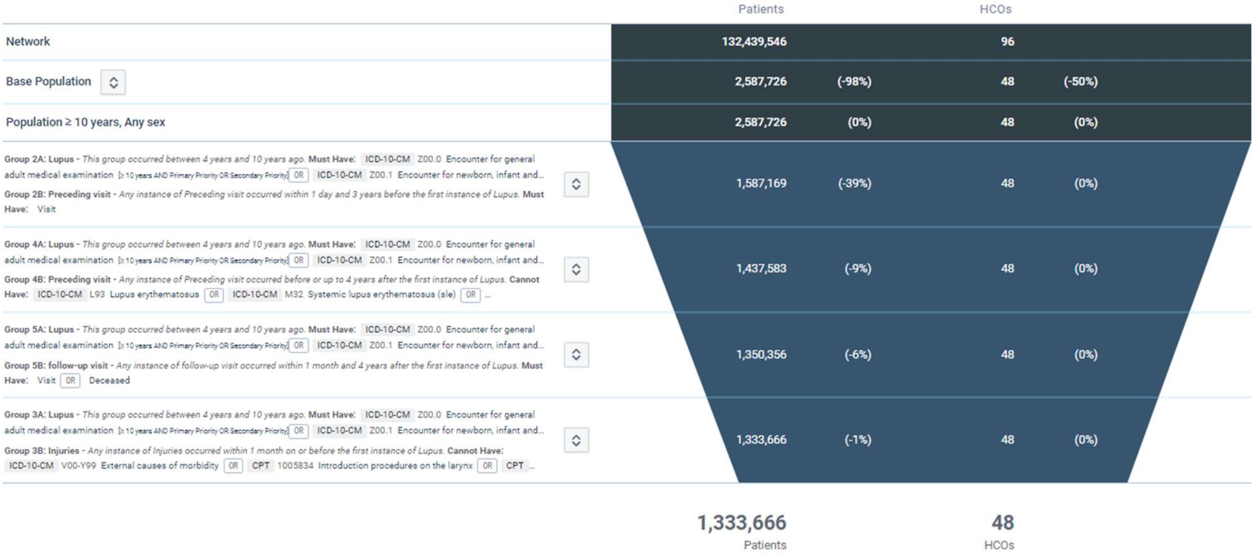


*Figure S3: Funnel diagram for the non-lupus cohort. The diagram illustrates the query groups described in the Supplemental File which are grouped according to similar time windows of assessment, indicating key International Classification of Diseases (ICD-10) codes, and ranked in terms of most to least impact on the sample size. The diagram illustrates the cohort's makeup prior to propensity matching and does not reflect the additional trimming that occurs when patients that do not match are discarded from the cohort. The number of healthcare organizations (HCOs) is also illustrated.*

*Table S1: Exclusion codes*

| Exclusions | Definition | Duration (days) |
|---|---|---|
| Diagnoses (ICD-10) | | |
| I72.6 | Aneurysm of vertebral artery | -∞ to 0 |
| I72.0 | Aneurysm of carotid artery | -∞ to 0 |
| I77.71 | Dissection of carotid artery | -∞ to 0 |
| I77.74 | Dissection of vertebral artery | -∞ to 0 |
| S15.0 | Injury of carotid artery of neck | -∞ to 0 |
| S15.1 | Injury of vertebral artery | -∞ to 0 |
| I77.7 | Other arterial dissection (including carotid, vertebral, basilar, and others) | -∞ to 0 |
| V00-Y99 | External causes of morbidity (e.g., motor vehicle accident, fall) | -30 to 0 |
| Procedures | | |
| 1004038 (CPT) | Surgical Procedures on the Spine (Vertebral Column) | -30 to 0 |
| 1005834 (CPT) | Introduction Procedures on the Larynx | -30 to 0 |
| 1022277 (CPT) | Transcatheter placement of extracranial vertebral artery stent(s), including radiologic supervision and interpretation, open or percutaneous | -∞ to 0 |
| 1022228 (CPT) | Transcatheter placement of intravascular stent(s), cervical carotid artery, open or percutaneous, including angioplasty, when performed, and radiological supervision and interpretation | -∞ to 0 |
| 35301 (CPT) | Thrombarterectomy, including patch graft, if performed; carotid, vertebral, subclavian, by neck incision | -∞ to 0 |
| 03QJ, 03QH, 03QN, 03QM, 03QL, 03QK (ICD-10-PCS) | Carotid artery repair | -∞ to 0 |
| 03QQ, 03QP (ICD-10-PCS) | Vertebral artery repair | -∞ to 0 |
| Non-lupus cohort only | | |
| D68.61 (ICD-10) | Antiphospholipid syndrome | -∞ to 730 |
| D68.62 (ICD-10) | Lupus anticoagulant syndrome | -∞ to 730 |
| H01.12 (ICD-10) | Discoid lupus erythematosus of eyelid | -∞ to 730 |
| L93 (ICD-10) | Lupus erythematosus (cutaneous) | -∞ to 730 |
| LG4652* | Nuclear antibody presence in serum, plasma, or blood | -∞ to 730 |
| LG350-5* | DNA double strand Ab [Units/volume] in serum, plasma, or blood. Filter: at least 15 IU/mL | -∞ to 730 |
| LG220-0* | Smith extractable nuclear Ab [Units/volume] in serum, plasma, or blood. Filter: at least 10 IU/mL | -∞ to 730 |
| M30-M36 (ICD-10) | Systemic connective tissue disorders | -∞ to 730 |
| M32 (ICD-10) | Systemic lupus erythematosus | -∞ to 730 |
| M05-M14 (ICD-10) | Inflammatory polyarthropathies | -∞ to 730 |
| Abbreviations: ∞ Exclusions over any time available in the preceding record; *custom TriNetX code; International Classification of Diseases, 10th Revision (ICD-10), ICD-10 Procedure Coding System (ICD-10-PCS) | | |

*Table S2: Propensity matched variables*

| **Variable** | **Description** | **Association with CeAD** | **Reference(s)** |
|---|---|---|---|
| Demographics | Age at index, current age, and sex | Varies | [2] |
| Diagnoses (ICD-10) | | | |
| J00-J06 | Acute upper respiratory infections | ⬆ | [3,4] |
| E06.3 | Autoimmune thyroiditis | ⬆ | [5] |
| E88.01 | Alpha-1-antitrypsin deficiency | ⬆ | [6] |
| Z55-Z65 | Adverse socioeconomic and psychosocial circumstances | ⬇ | [3,7] |
| I71 | Aortic aneurysm and dissection | ⬆ | [8] |
| I77.3 | Arterial fibromuscular dysplasia | ⬆ | [9] |
| E08-E13 | Diabetes mellitus | ⬇ | [10–12] |
| I70-I79 | Diseases of arteries, arterioles, and capillaries (includes aneurysms, arterial embolism, arteritis, and arterial fibromuscular dysplasia) | ⬆ | [8,9] |
| Q79.6 | Ehlers-Danlos syndromes | ⬆ | [9] |
| Z82.4 | Family history of ischemic heart disease and other diseases of the circulatory system | ⬆ | [10] |
| E72.11 | Homocystinuria | ⬆ | [13] |
| E78.5 | Hyperlipidemia, unspecified | ⬇ | [10,11,14] |
| I10-I1A | Hypertensive diseases | ⬆ | [10] |
| F10-F19 | Mental and behavioral disorders due to psychoactive substance use | ⬆ | [15,16] |
| G43 | Migraine | ⬆ | [10] |
| Q78.0 | Osteogenesis imperfecta | ⬆ | [9] |
| Q87 | Other specified congenital syndromes (includes Marfan syndrome, Loeys Dietz syndrome, Alport syndrome, arterial tortuosity syndrome) | ⬆ | [9,17] |
| E66 | Overweight and obesity | ⬇ | [10] |
| O00-O9A | Pregnancy, childbirth and the puerperium | ⬆ | [18] |
| Z72.0 | Tobacco use | ⬆ | [10,11] |
| Procedures/treatments | | | |
| CV490 (VA) | Antihypertensives, other | ⬇ | [10,14] |
| CN105 (VA) | Antimigraine agents (includes triptans) | ⬆ | [19] |
| CV100 (VA) | Beta blockers/related | ⬇ | [10] |
| HS200 (VA) | Contraceptives, systemic | ⬆ | [20] |
| AM400 (VA) | Quinolones (includes fluoroquinolones) | ⬆ | [21] |
| Abbreviations: International Classification of Diseases, 10th Edition (ICD-10), Veterans Affairs National Drug File (VA), positive association (⬆), negative association (⬇) | | | |

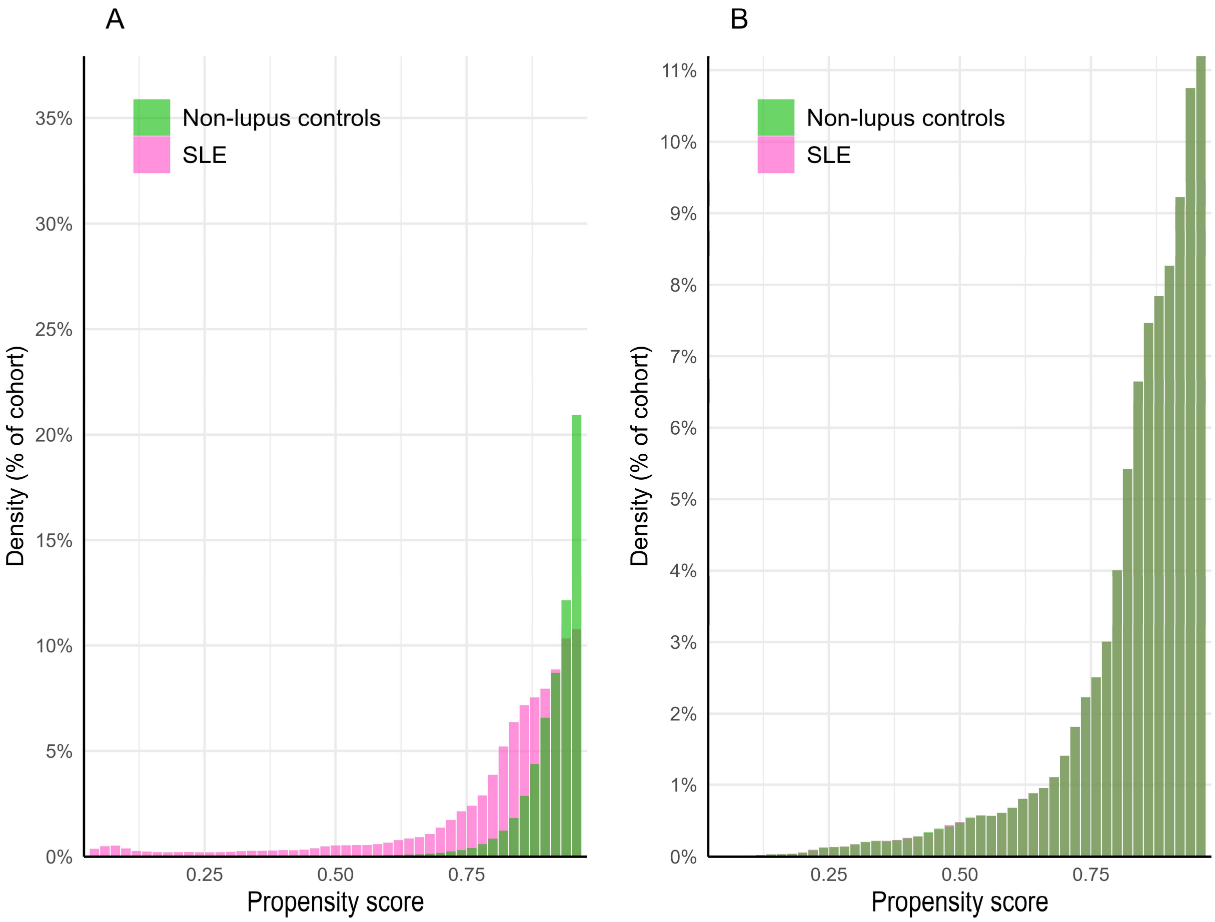


*Figure S4: Propensity score density graph. Density scores before (A) and after (B) matching. The pink bars indicate the systemic lupus erythematosus (SLE) cohort while the green bars represent the non-lupus control cohort. After matching, densities overlap closely suggesting sufficient balance of covariates.*

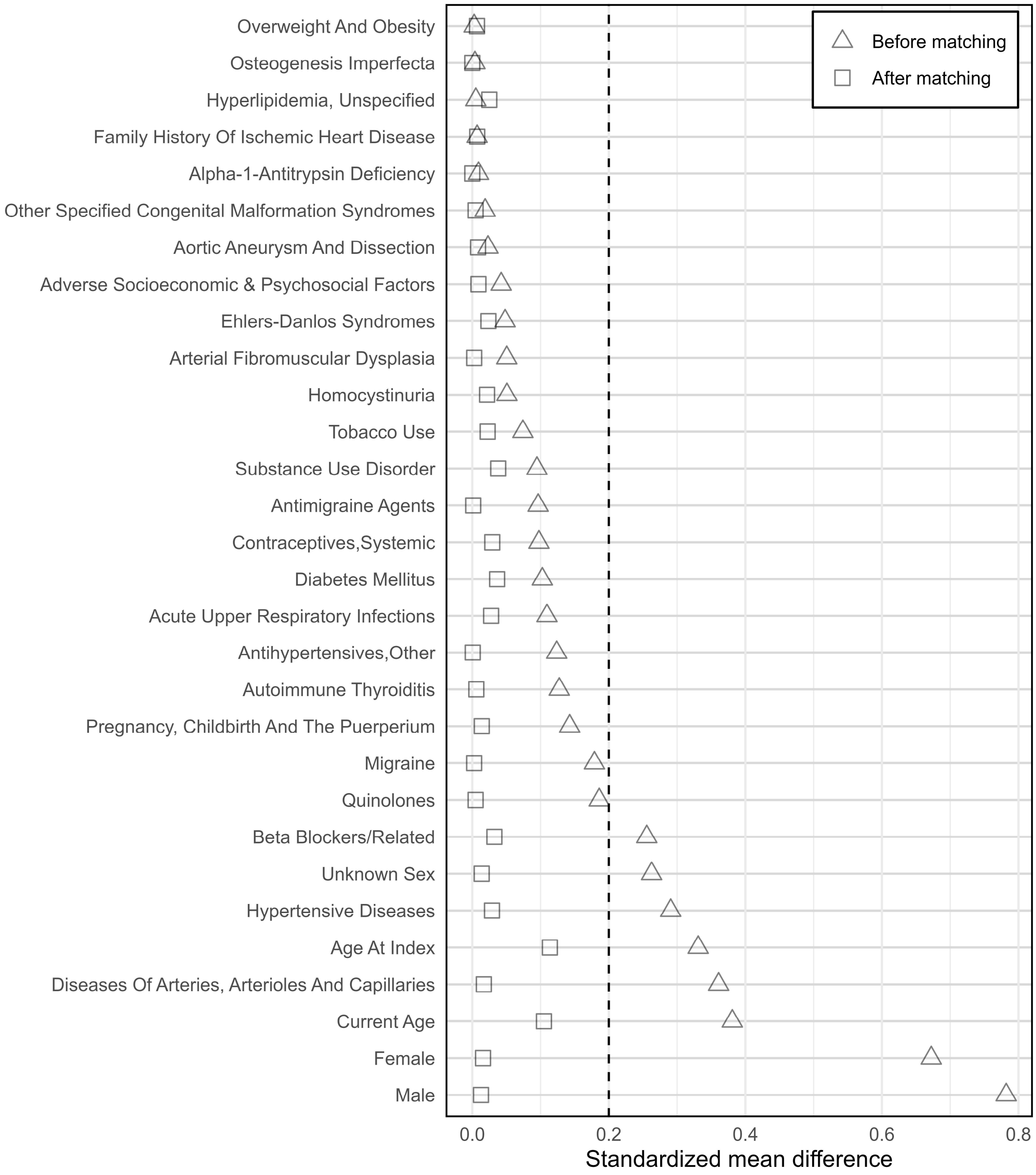


*Figure S5: Covariate balance (Love) plot. Standardized mean differences (SMDs) between cohorts before and after propensity score matching are shown. The vertical dashed line at SMD=0.2 represents the threshold for acceptable covariate balance [22,23]. Triangles indicate SMDs before matching for each covariate, while squares show SMDs after matching. This plot demonstrates the improvement in covariate balance through propensity score matching, with all covariates ultimately having adequate balance after matching. Plot created by Robert J. Trager using R and R studio (version 4.2.2, Vienna, AT [24]) and the ggplot2 package [25].*

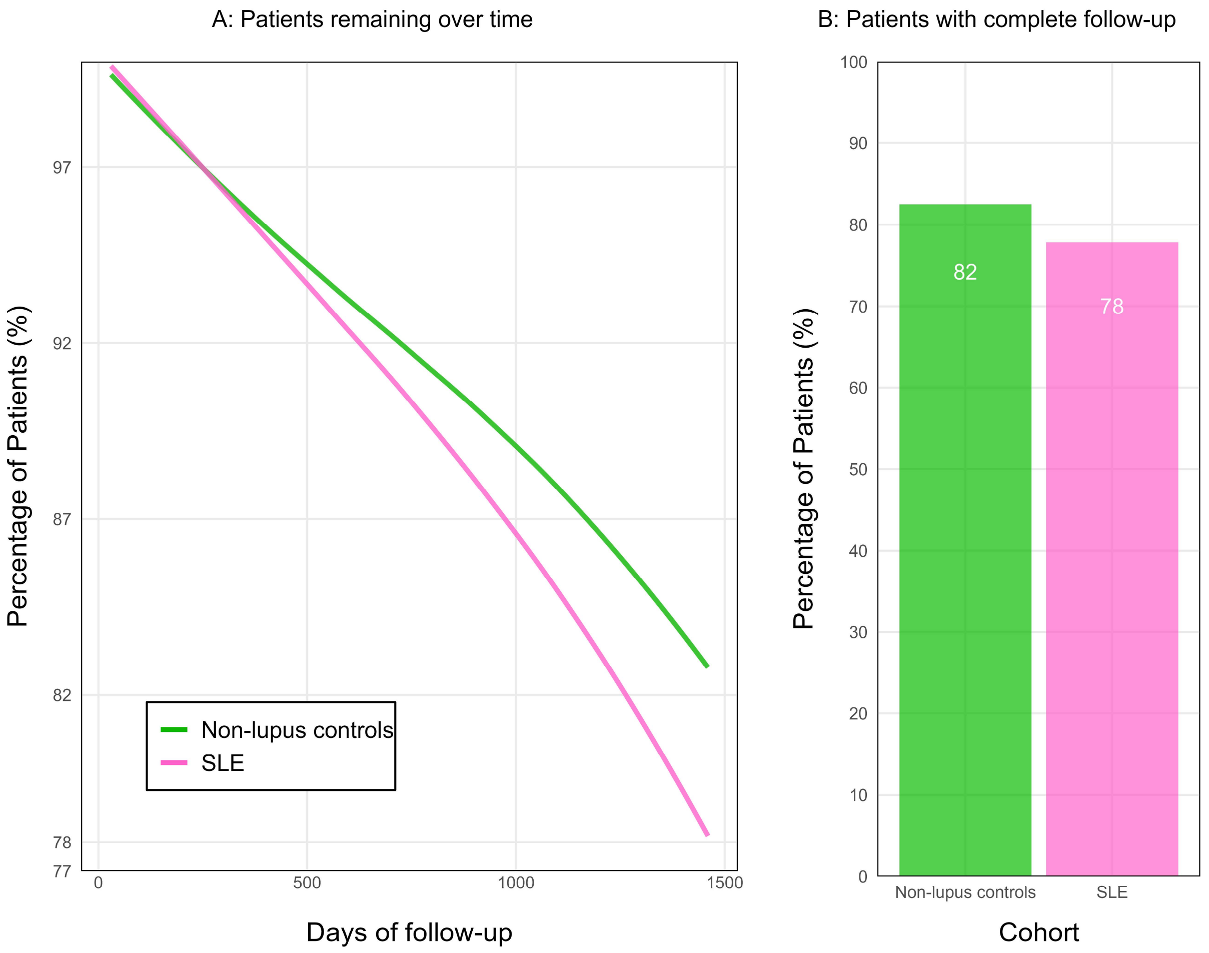


*Figure S6. Follow-up data. A: This plot illustrates the percentage of patients remaining in each cohort throughout the duration of follow-up, indicating the systemic lupus erythematosus (SLE) cohort in pink and non-lupus controls in green. This plot incorporates locally estimated scatterplot smoothing. B: The bar plot displays the percentage of patients who remained in each cohort for at least the maximum follow-up time available (i.e., Non-lupus: 82%; SLE: 78%). Both plots were created by Robert J. Trager using R and R studio (version 4.2.2, Vienna, AT [24]) and the ggplot2 package [25].*

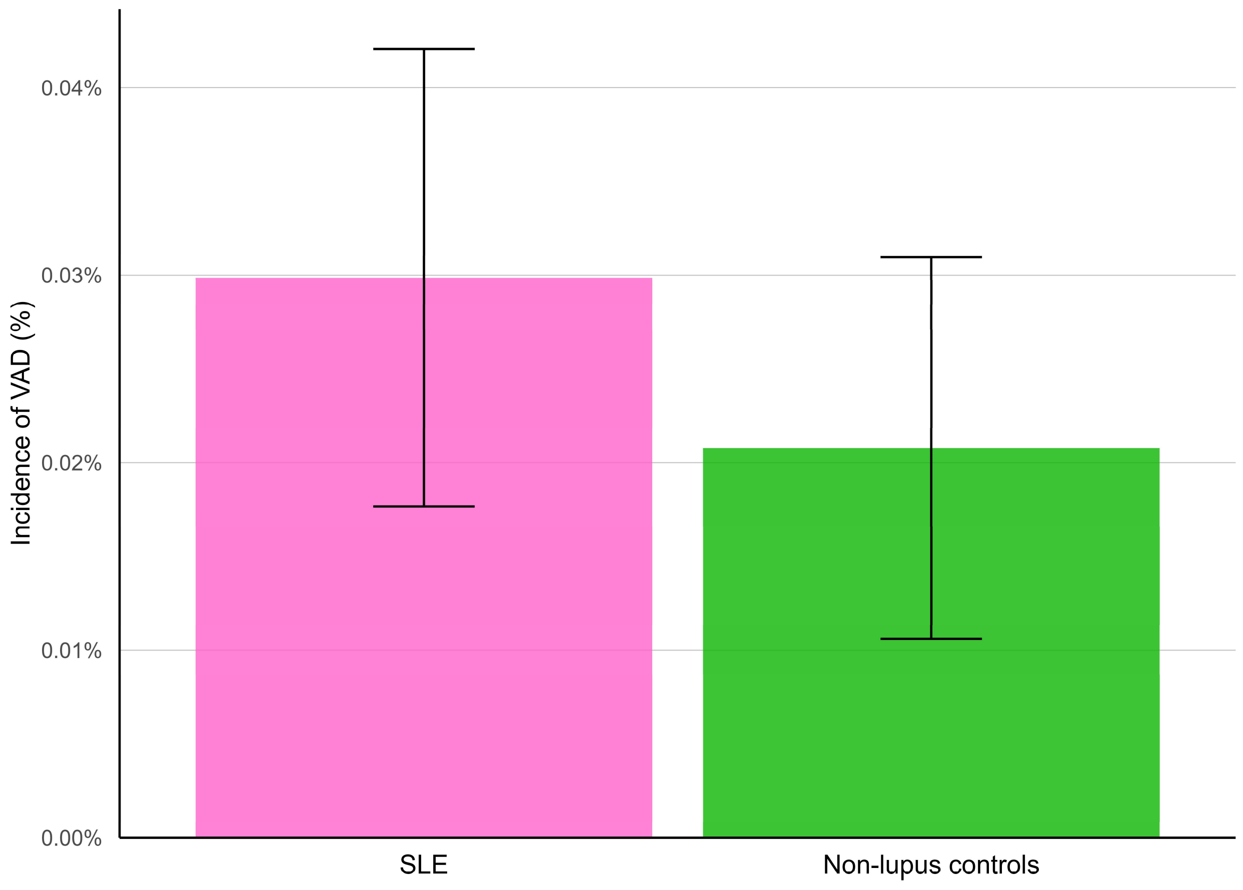


*Figure S7: Total incidence of vertebral artery dissection (VAD) over the four-year follow-up window. Incidences of VAD are shown for the systemic lupus erythematosus cohort (SLE; pink) and non-lupus controls (green). Brackets indicate 95% confidence intervals.*

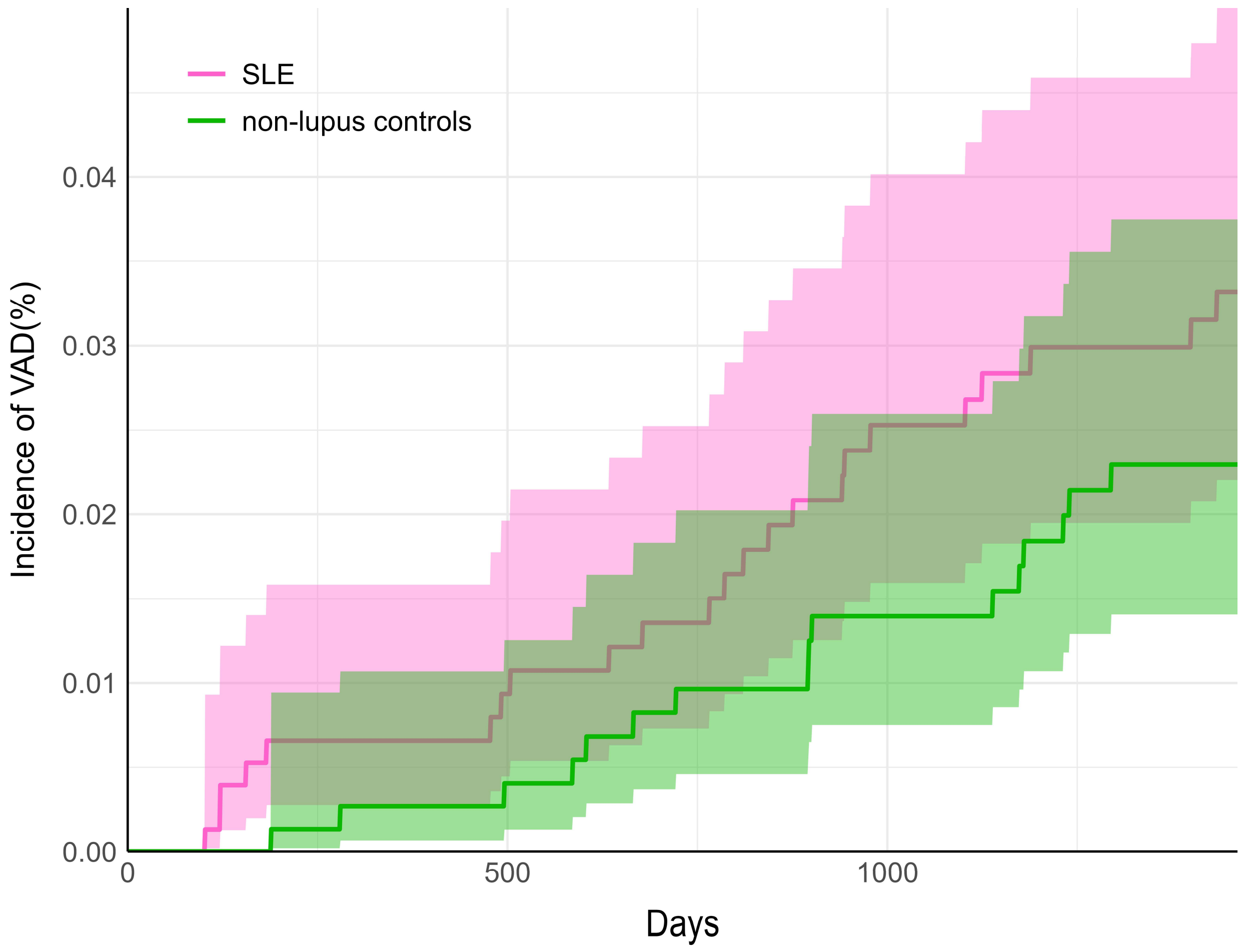


*Figure S8: Cumulative incidence of vertebral artery dissection (VAD). Incidences of CeAD in the systemic lupus erythematosus cohort (SLE; pink) and non-lupus controls (green) are illustrated over the four-year follow-up period (1460 days). Shaded regions indicate 95% confidence intervals.*

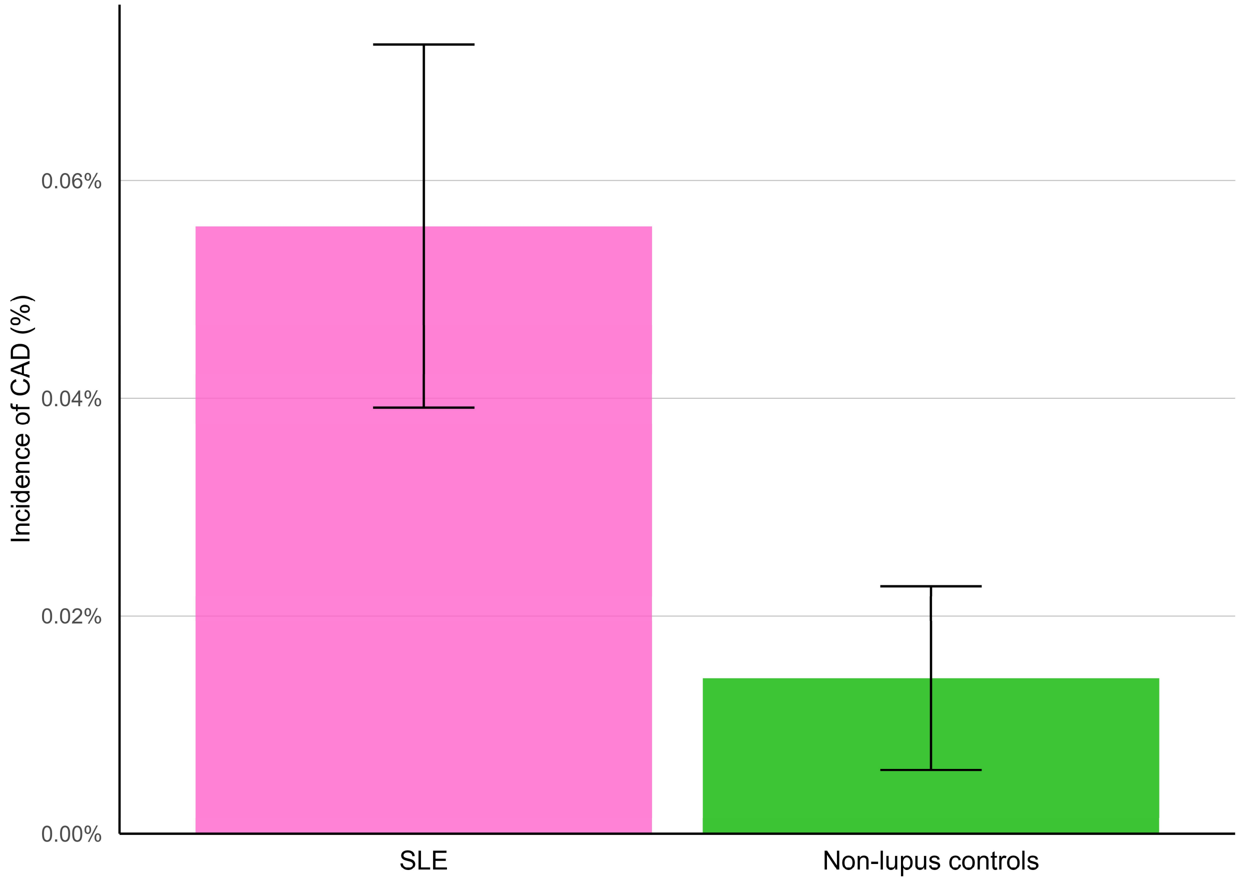


*Figure S9: Total incidence of carotid artery dissection (CAD) over the four-year follow-up window. Incidences of CAD are shown for the systemic lupus erythematosus cohort (SLE; pink) and non-lupus controls (green). Brackets indicate 95% confidence intervals.*

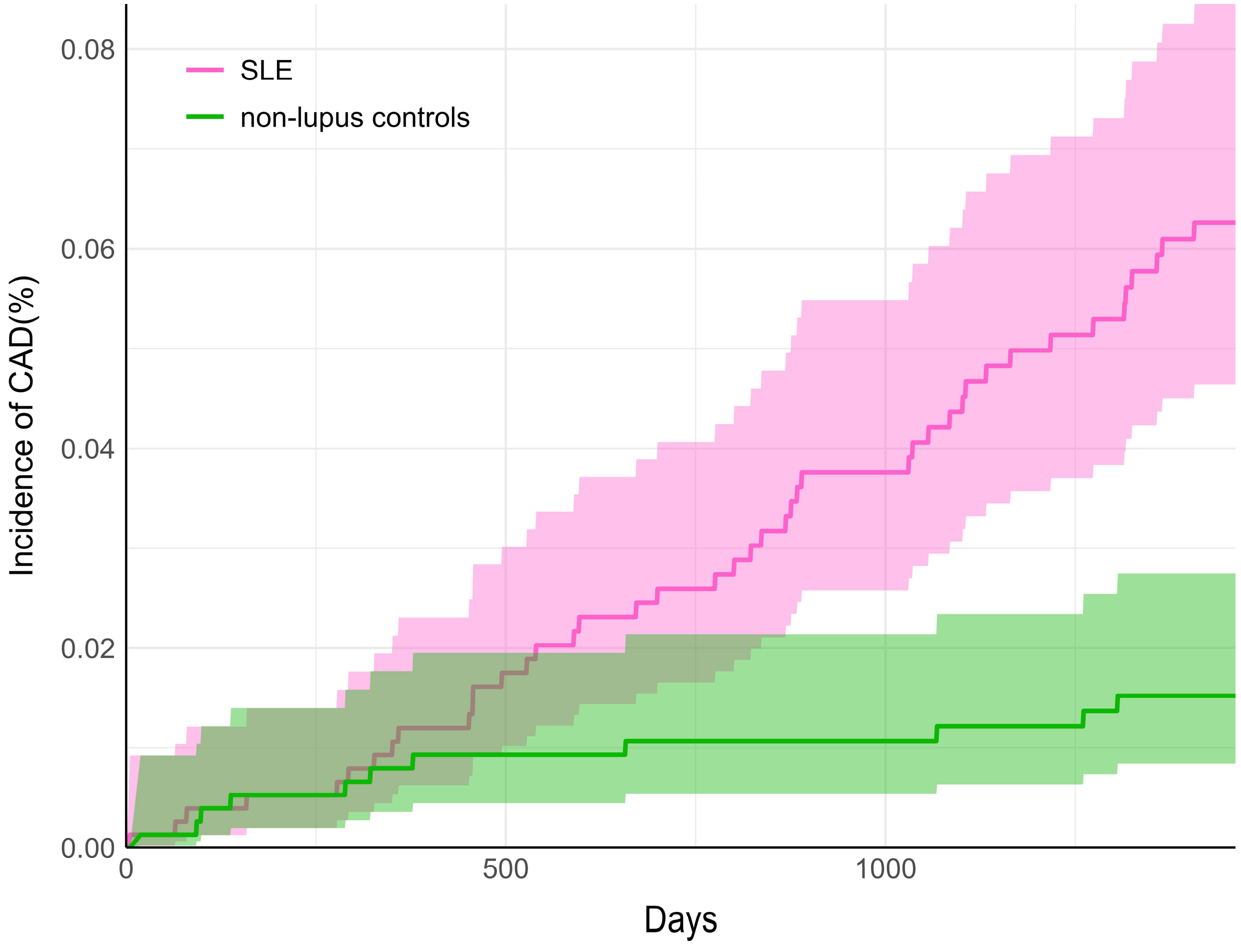


*Figure S10: Cumulative incidence of carotid artery dissection (CAD). Incidences of CeAD in the systemic lupus erythematosus cohort (SLE; pink) and non-lupus controls (green) are illustrated over the four-year follow-up period (1460 days). Shaded regions indicate 95% confidence intervals.*

# References


1. Wang SV, Schneeweiss S: A Framework for Visualizing Study Designs and Data Observability in Electronic Health Record Data. Clin Epidemiol. 2022, 14:601–8. 10.2147/CLEP.S358583

2. Griffin KJ, Harmsen WS, Mandrekar J, Brown RD, Keser Z: Epidemiology of Spontaneous Cervical Artery Dissection: Population-Based Study. Stroke. 2024, 55:670–7. 10.1161/STROKEAHA.123.043647

3. Grau AJ, Brandt T, Buggle F, et al.: Association of Cervical Artery Dissection With Recent Infection. Archives of Neurology. 1999, 56:851–6. 10.1001/archneur.56.7.851

4. Witsch J, Rutrick SB, Lansdale KN, et al.: Influenza-Like Illness as a Short-Term Risk Factor for Arterial Dissection. Stroke. 2023, 54:e66–8. 10.1161/STROKEAHA.122.042367

5. Pezzini A, Del Zotto E, Mazziotti G, et al.: Thyroid Autoimmunity and Spontaneous Cervical Artery Dissection. Stroke. 2006, 37:2375–7. 10.1161/01.STR.0000236500.15976.f3

6. Pezzini A, Magoni M, Corda L, et al.: Alpha-1-antitrypsin deficiency-associated cervical artery dissection: report of three cases. Eur Neurol. 2002, 47:201–4. 10.1159/000057899

7. Kellert L, Grau A, Pezzini A, et al.: University education and cervical artery dissection. J Neurol. 2018, 265:1065–70. 10.1007/s00415-018-8798-7

8. Witsch J, Mir SA, Parikh NS, et al.: Association Between Cervical Artery Dissection and Aortic Dissection. Circulation. 2021, 144:840–2. 10.1161/CIRCULATIONAHA.121.055274

9. Debette S: Pathophysiology and risk factors of cervical artery dissection: what have we learnt from large hospital-based cohorts? Current Opinion in Neurology. 2014, 27:20–8. 10.1097/WCO.0000000000000056

10. Del Zotto E, Grassi M, Zedde M, et al.: Risk profile of patients with spontaneous cervical artery dissection. Annals of Neurology. 2023, 94:585–95.

11. Abdelnour LH, Abdalla ME, Elhassan S, Kheirelseid EAH: Meta-analysis of cardiovascular risk profile of stroke secondary to spontaneous cervical artery dissection compared to ischemic stroke of other causes. Health Sciences Review. 2022, 5:100058. 10.1016/j.hsr.2022.100058

12. Engelter ST, Grond-Ginsbach C, Metso TM, et al.: Cervical artery dissection: trauma and other potential mechanical trigger events. Neurology. 2013, 80:1950–7. 10.1212/WNL.0b013e318293e2eb

13. Gallai V, Caso V, Paciaroni M, Cardaioli G, Arning E, Bottiglieri T, Parnetti L: Mild Hyperhomocyst(e)inemia. Stroke. 2001, 32:714–8. 10.1161/01.STR.32.3.714

14. Abdelnour LH, Abdalla ME, Elhassan S, Kheirelseid EAH: Diabetes, hypertension, smoking, and hyperlipidemia as risk factors for spontaneous cervical artery dissection: Meta-analysis of case-control studies. Current Journal of Neurology. 2022, 21:183–93. 10.18502/cjn.v21i3.11112

15. Hori S, Hori E, Umemura K, et al.: Anatomical Variations of Vertebrobasilar Artery are Closely Related to the Occurrence of Vertebral Artery Dissection—An MR Angiography Study. Journal of Stroke and Cerebrovascular Diseases. 2020, 29:104636. 10.1016/j.jstrokecerebrovasdis.2020.104636

16. Smith WS, Johnston SC, Skalabrin EJ, Weaver M, Azari P, Albers GW, Gress DR: Spinal manipulative therapy is an independent risk factor for vertebral artery dissection. Neurology. 2003, 60:1424–8. 10.1212/01.WNL.0000063305.61050.E6

17. Traenka C, Kloss M, Strom T, et al.: Rare genetic variants in patients with cervical artery dissection. Eur Stroke J. 2019, 4:355–62. 10.1177/2396987319861869

18. Trager RJ, Daniels CJ, Scott ZE, Perez JA: Pregnancy and spontaneous cervical artery dissection: A propensity-matched retrospective cohort study. Journal of Stroke and Cerebrovascular Diseases. 2023, 32:107384. 10.1016/j.jstrokecerebrovasdis.2023.107384

19. Roberto G, Piccinni C, D'Alessandro R, Poluzzi E: Triptans and serious adverse vascular events: Data mining of the FDA Adverse Event Reporting System database. Cephalalgia. 2014, 34:5–13. 10.1177/0333102413499649

20. D'Anglejan-Chatillon J, Ribeiro V, Mas J l., Youl B d., Bousser M g.: Migraine - A Risk Factor for Dissection of Cervical Arteries. Headache: The Journal of Head and Face Pain. 1989, 29:560–1. 10.1111/j.1526-4610.1989.hed2909560.x

21. Zotto ED, Pezzini A: Use of fluoroquinolones and the risk of spontaneous cervical artery dissection. Eur J Neurol. 2019, 26:1028–31. https://doi.org/10.1111/ene.13917

22. Austin PC: Balance diagnostics for comparing the distribution of baseline covariates between treatment groups in propensity-score matched samples. Statistics in Medicine. 2009, 28:3083–107. 10.1002/sim.3697

23. Stuart EA, Lee BK, Leacy FP: Prognostic score–based balance measures can be a useful diagnostic for propensity score methods in comparative effectiveness research. Journal of Clinical Epidemiology. 2013, 66:S84-S90.e1. 10.1016/j.jclinepi.2013.01.013

24. R Core Team: R: A Language and Environment for Statistical Computing. R Foundation for Statistical Computing: Vienna, Austria; 2022.

25. Wickham H: ggplot2: Elegant Graphics for Data Analysis. Springer-Verlag New York; 2016.
